# Crippled Soldiers and Sailors to Be Taught Useful Occupations

**Published:** 1918-12

**Authors:** 


### Crippled Soldiers and Sailors to Be Taught Useful Occupations.

American soldiers and sailors crippled in the war are to be given every opportunity, in addition to war risk insurance indemnity and pensions, to learn new trades or professions in order that they may resume their place of usefulness in civil life without the handicaps that ordinarily surround a man deprived of arms, legs, sight or hearing.

One of the most interesting institutions that has been established here as a result of the war is the Red Cross institute for Crippled and Disabled Men at 311 Fourth avenue, where four schools, the nuclei of others that are, it is said, to be organized in Chicago, St. Louis and other places, are now under way teaching cripples the manufacture of artificial limbs, linotype and monotype operating, mechanical drafting and oxyceteline welding. These four local schools have a teaching capacity for 300 men.

The recent passage by congress of the Smith-Sears bill, providing an appropriation of $2,000,000 to be used in the discretion of the Federal Board of Vocational Education contemplated, it is said, the elaboration of the plans which are now being worked out in this city. Soon, it is thought, there will be additional schools in many other parts of the United States they were in fields that were not overcrowded. A soldier or sailor, for instance, instead of having to pay $150 to $200 for an artificial limb may procure one for $30 and, if he desires, be taught how to make it himself, at the same time acquiring a trade that will pay him $4 to $8 a day.

Frank R. Bigler, a cripple for thirty-one years and minus a leg and an arm, is the industrial agent of the institue, which was founded about a year ago by Jeremiah Milbank with an initial endowment of $50,000 and a building in which to carry on its work. Mr. Bigler came from Kansas City, Mo., his services being loaned by an industrial corporation there, to instill optimism and good cheer into the minds of all returning war cripples.

Douglas C. McMurtrie, director of the institute, a graduate of the Massachusetts Institute of Technology and in charge of the department of printing at Columbia University, has made a study of the social and economic reconstruction of cripples for more than eight years.

Mr. McMurtrie made it clear that while the institute has no official arrangement with the government authorities relative to the utilization of its facilites in the rehabiltation of war cripples, that department being in charge of Surgeon General William C. Gorgas, when soldiers and sailors are discharged from army and navy hospitals then the institute will offer them every chance to "come back." Positions will be found for the men and, where they are unable to pay expenses while learning their new trade, funds will be advanced them to continue their training until competent to take a job.

The institute, however, is a national activity of the American Red Cross, responsible to the War Council through the Director General of Military Relief, Jesse H. Jones. It is the only non-commercial institution of the kind in the United States, says Mr. McMurtrie, and its purpose is broadly humanitarian, taking in civilian as well as military cripples. It is not a charitable institution but intended to be self-supporting.

"Already the natonal authorities have gone on record," said Mr. McMurtrie, "as accepting without reservation responsibility for the after-care of men injured in the service. The surgeon-general's office of the war department is now preparing to provide for wounded men, not only medical and surgical care, but also the curative advances afforded by the simpler forms of occupation. The government is further inaugurating vocational training having as its object rehabilitation for self-support. The government, however, is disposed to make use of, under the supervision, such private assistance as may be offered and found of value."

